# Cataract surgery in patients with Fuchs’ dystrophy and corneal decompensation indicated for Descemet's membrane endothelial keratoplasty

**DOI:** 10.1038/s41598-022-12434-8

**Published:** 2022-05-19

**Authors:** Wei-Yi Chou, Yih-Shiuan Kuo, Pei-Yu Lin

**Affiliations:** 1 Department of Ophthalmology, Taipei Veterans General Hospital, 201, Sec. 2, ShihPai Rd., Taipei, Taiwan 11217; 2 Department of Ophthalmology, Zhongxing Branch, Taipei City Hospital, Taipei, Taiwan; 3 Faculty of Medicine, School of Medicine, National Yang Ming Chiao Tung University, Taipei, Taiwan

**Keywords:** Outcomes research, Optical materials and structures

## Abstract

The availability of corneal donor tissue is limited in most developing countries. This study evaluated whether patients with coexisting cataract and Fuchs’ dystrophy with corneal decompensation awaiting Descemet’s membrane endothelial keratoplasty (DMEK) benefited from phacoemulsification. This is a retrospective case–control study which included patients with Fuchs’ dystrophy and evidence of corneal decompensation awaiting DMEK. Best-corrected visual acuity (BCVA) and central corneal thickness (CCT) were documented at baseline (pre-cataract surgery in the case group, or at the time of transplantation registry in the control group), 1-month and pre-DMEK. A total of 16 phakic patients with visually significant cataracts had cataract surgery during the study period, and 15 pseudophakic patients were included as controls. There was no significant difference with regard to BCVA at baseline, 1-month or pre-DMEK between the case and control groups. Similarly, no significant difference in CCT was found at baseline, 1-month or pre-DMEK. In the case group, 4 patients with improved visual acuity post-cataract surgery chose to defer DMEK. After stratification, statistical analysis showed significantly better BCVA in the deferred group (*n* = 4) at 1-month post-cataract surgery, compared to the DMEK group (*n* = 12) (0.21 ± 0.21 vs. 0.86 ± 0.29 LogMAR, *P* = 0.004). The other parameters, including baseline BCVA and CCT at any time point documented, were not statistically different. In conclusion, in patients with Fuchs’ dystrophy and decompensated corneas awaiting transplantation, phacoemulsification did not lead to significant increase of corneal thickness nor deterioration of visual acuity. A few patients achieved satisfactory vision after cataract surgery and deferred endothelial keratoplasty.

## Introduction

Corneal endothelial cells maintain transparency of the cornea by actively pumping fluid out of the tissue. As endothelial function debilitates to the point unable to maintain corneal deturgescence, visual acuity declines^1,2^. Common causes of corneal endothelial dysfunction include Fuchs’ endothelial corneal dystrophy (FECD), surgical or laser trauma, and post-infectious endothelial dysfunction^2^. FECD is an autosomal dominant disease of the corneal endothelium characterized by progressive endothelial cell loss with formation of posterior focal excrescences termed guttae^3^; it is also the most common indication for corneal transplantation worldwide^4^. When corneal edema and visually significant cataract coexist, the surgeon faces the dilemma of whether to perform cataract surgery or not. Phacoemulsification has been shown to decrease endothelial cells by 8.5–16%^5,6^. Factors such as a low endothelial cell count, the presence of stromal edema, guttae, and epithelial microcystic changes indicate an increased possibility for corneal decompensation after cataract surgery^7^. Performing cataract surgery in patients with preexisting corneal decompensation risks further increase in corneal edema and deterioration of visual acuity. Previously, triple Descemet's membrane endothelial keratoplasty (DMEK combined with phacoemulsification and posterior chamber lens implantation) was recommended to treat such patients, due to rapid visual recovery and the advantage of a 1-stage procedure with reduced risks^7,8^. However, eye banks cannot match demand worldwide, and the availability of corneal donor tissue is limited in most developing countries, with the majority of patients never receiving a graft^4,9^. According to a global survey by Gain et al*.*^4^, around 12.7 million people were awaiting transplantation, and the mismatch between supply and demand was a staggering ratio of 1:70. Correspondingly, the Taiwan Organ Registry and Sharing Center reported that 617 corneal transplantations were performed in 2020, with 912 patients still on the waiting list (as of Dec 26th, 2020)^10^. With the advance of surgical techniques, FECD with corneal decompensation can now be successfully treated by endothelial keratoplasty or triple procedure; however, donor shortages remain an issue^11^, and cataract surgery alone could be a traditional yet easily accessible intervention conducive to visual rehabilitation. Therefore, the primary aim of this study was to assess whether FECD patients with corneal decompensation and concurrent visually significant cataract achieved visual improvement after receiving phacoemulsification, while awaiting endothelial keratoplasty.

## Methods

**Figure 1:**
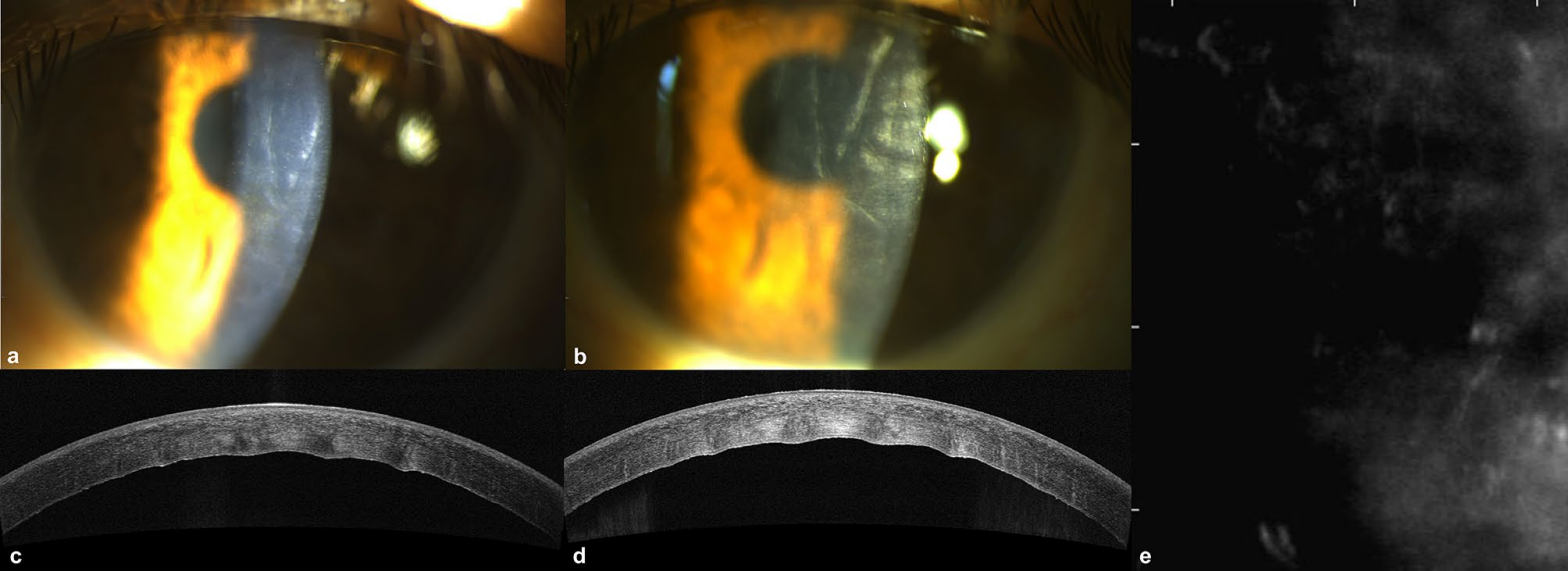
(**a**) External eye photography and (**c**) anterior-segment OCT in a patient with Fuchs’ dystrophy and corneal edema before phacoemulsification surgery. (**b**, **d**) The same patient after surgery. (**e**) Specular microscopy showed indecipherable endothelial cell density before surgery.

This research was designed as a single-institution, retrospective, case–control study. Data collection and chart review were approved by the Institutional Review Board of Taipei Veterans General Hospital (IRB-TPEVGH No.2021-06-006CC). Due to the retrospective design, the need for informed consent was waived by the Institutional Review Board of Taipei Veterans General Hospital. The study was conducted in accordance with the tenets of the Declaration of Helsinki. Records of patients with FECD awaiting DMEK from January 2018 to September 2020 were examined. All patients had undergone comprehensive ophthalmic examinations by corneal specialists, including best-corrected visual acuity, intraocular pressure measurement, slit-lamp biomicroscopy and dilated fundus examination. FECD disease severity was ranked according to the Krachmer grading scale^12^. Since guttae first appear in the center of the cornea^12,13^, corneal edema in FECD presents centrally while edema from other etiologies are usually diffuse. Therefore, there was no adequate universal grading for corneal edema caused by FECD. Under this consideration, we had adapted the Oxford Cataract Treatment and Evaluation Team (OCTET)^14^ grading for documentation purposes. The OCTET grades transient corneal edema with varying degrees of Descemet membrane folds. Aside from the OCTET grading, persistent edema was individually documented. All study participants met inclusion criteria as follows: (a) FECD Krachmer grade 5, defined as greater than 5-mm confluent central guttae with stromal or epithelial edema^12^; (b) preoperative slit-lamp evidence of corneal decompensation, defined as corneal edema due to endothelial loss (Fig. 1e), and manifested by persistent stromal edema, stromal haze and Descemet membrane folds (Fig. 1a). Patients in the case group had visually significant cataracts and received cataract surgery during the study period. Figure 1 demonstrates a typical patient before (Fig. 1a, c, e) and after (Fig. 1b, d) cataract surgery. Patients in the control group were pseudophakic, having undergone prior cataract surgery more than 1 year before enrollment (ranging from 1 to 20 years). Subjects were excluded from the study if they had non-visually significant cataracts, other causes of corneal edema apart from FECD (including but not limited to pseudophakic bullous keratopathy), or concurrent ocular comorbidity, such as glaucoma and age-related macular degeneration.

Collected data included cataract grading, best-corrected visual acuity (BCVA) and central corneal thickness (CCT). Cataract severity was documented according to the Lens Opacities Classification System III (LOCS III)^15^. BCVA was documented at baseline (pre-cataract surgery in the case group, or at the time of registry for transplantation in the control group), 1 month (post-cataract surgery in the case group, or post-registry in the control group), and pre-DMEK (or at 6 months post-cataract surgery if the patient did not receive DMEK). Similarly, CCT was measured at baseline, 1-month, and pre-DMEK (or at 6 months post-cataract surgery if the patient did not receive DMEK). The interval between cataract surgery and DMEK in the case group was also compared to the interval between transplantation registries to DMEK in the control group.

Optical coherence tomography (OCT) scans were performed using the RTVue XR Avanti SD-OCT (Optovue, Inc. Fremont, CA, USA). Precise measurements of CCT were obtained from SD-OCT generated pachymetry maps and manually measured by an experienced ophthalmic technician if not produced automatically.

Phacoemulsification and aspiration were performed by a single experienced surgeon (P.Y.L), using a combination of topical and subconjunctival anesthesia. Incisions were made at the anatomic limbus, with tunneling into clear cornea. The technique of phacoemulsification was “stop and chop,” with special care to keep the phacoemulsification tip pointed away from the endothelium. Intraoperative ophthalmic viscosurgical device (OVD) was a cohesive OVD. The surgical techniques were standardized to reflect best practice by the surgeon.

Visual acuity data were measured using the Snellen chart and converted to logarithm of the minimum angle of resolution (logMAR) scale for analysis and averaging. Nonparametric tests were used for the following comparisons because most measurement data were not normally distributed. Categorical variables were analyzed using chi-square tests. Measurements were compared between the case and control groups, and subsequent DMEK and non-DMEK groups, using Mann–Whitney *U* tests. Measurements were compared between baseline and pre-DMEK CCT in either group using Wilcoxon signed rank test. To evaluate the ability of baseline BCVA to discriminate between DMEK and non-DMEK eyes, we calculated the area under the receiver operating characteristic curve (AUROC). Statistical significance was defined as *P* ≤ 0.05 and all analyses were conducted using SPSS statistical software (version 22, SPSS Inc., Chicago, IL, USA).

## Results

### Patient population

The case group comprised 16 phakic patients who received cataract surgery while awaiting DMEK, and the control group had 15 pseudophakic patients who were also on the DMEK waiting list. The average age was 68.53 ± 10.49 years in the control group and 68.00 ± 7.82 years in the case group (*P* = 0.545, Table 1). The case group had a significantly larger proportion of female patients (*P* = 0.044). The interval between transplantation registry to DMEK in the control group was 135.73 ± 73.06 days, and the interval between cataract surgery and DMEK in the case group was 168.58 ± 83.15 days, this difference being insignificant (*P* = 0.277). The baseline BCVA in the control and case groups was 0.70 ± 0.43 logMAR and 0.63 ± 0.30 logMAR, respectively (*P* = 0.626). The baseline CCT in the control and case groups was 690.53 ± 106.74 µm and 649.19 ± 54.65 µm, respectively (*P* = 0.338).

**Table 1:** Baseline Characteristics.

	Control group (*n* = 15)^a^	Case group (*n* = 16)^a^	*P*
Age (years)	68.53 ± 10.49	68.00 ± 7.82	0.545
Female, % (*n*)	46.7 (7)	81.3 (13)	0.044*
Interval (days)^b^	135.73 ± 73.06	168.58 ± 83.15	0.277
BCVA at baseline (logMAR)	0.70 ± 0.43	0.63 ± 0.30	0.626
CCT at baseline (µm)	690.53 ± 106.74	649.19 ± 54.65	0.338

^a^All values are presented as mean ± SD unless otherwise noted.

^b^Interval defined as time between transplantation registry to DMEK in the control group, and time between cataract surgery and DMEK in the case group.

BCVA, best corrected visual acuity; CCT, central corneal thickness.

### Postoperative data

There was no statistical difference with regard to BCVA at 1-month or pre-DMEK between the control and case groups (Table 2, both *P* > 0.05). Similarly, at 1-month after cataract surgery, CCT in the case group was not significantly different compared to the control group (645.12 ± 70.13 µm vs. 679.11 ± 100.12 µm, *P* = 0.393), and also returned to baseline value. Before DMEK, no significant difference in pachymetry was found between the two groups, either (*P* = 0.470). In addition, no statistical difference was observed between baseline and pre-DMEK CCT in either the case group (649.19 ± 54.65 vs. 664.50 ± 98.37, *P* = 0.569) or the control group (690.53 ± 106.74 vs. 676.80 ± 82.19, *P* = 0.507, data not shown).

**Table 2:** BCVA and CCT in the control and case groups.

	Control group (*n* = 15)^a^	Case group (*n* = 16)^a^	*P*^b^
BCVA at baseline (logMAR)	0.70 ± 0.43	0.63 ± 0.30	0.626
BCVA at 1 month (logMAR)	0.65 ± 0.41	0.70 ± 0.40	0.401
BCVA pre-DMEK^c^ (logMAR)	0.68 ± 0.43	0.63 ± 0.44	0.800
BCVA change pre-DMEK^c^ (logMAR)	− 0.02 ± 0.23	0.00 ± 0.47	0.711
CCT at baseline (µm)	690.53 ± 106.74	649.19 ± 54.65	0.338
CCT at 1 month (µm)	679.11 ± 100.12	645.12 ± 70.13	0.393
CCT pre-DMEK^c^ (µm)	676.80 ± 82.19	664.50 ± 98.37	0.470
CCT change pre-DMEK^c^ (µm)	− 13.73 ± 52.67	15.31 ± 77.78	0.379

^a^All values are presented as mean ± SD unless otherwise noted.

^b^Mann-Whitney *U* test.

^c^CCT and BCVA documented at 6 months if DMEK surgery was deferred.

BCVA, best corrected visual acuity; CCT, central corneal thickness; DMEK, Descemet’s membrane endothelial keratoplasty.

### Subgroup analysis

**Figure 2:**
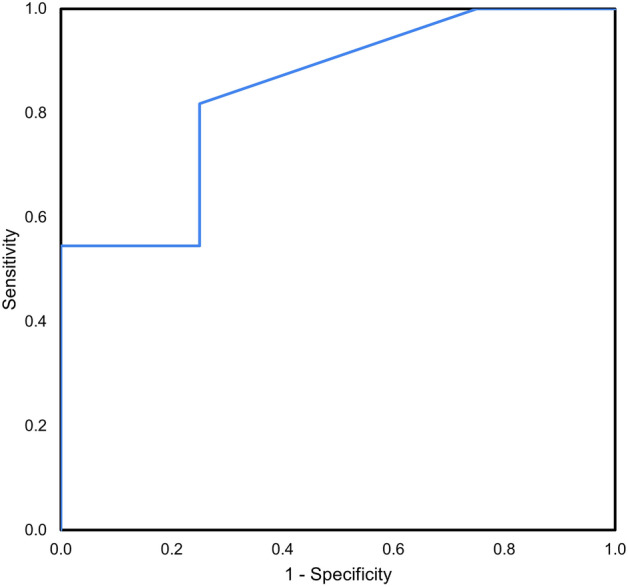
Area under receiver operating characteristics curve (AUROC) of prediction for progression to DMEK (AUROC = 0.841).

In the case group, there were 4 patients (25%) with improved visual acuity after cataract surgery who chose to defer DMEK. Thus, we stratified the case group into those who subsequently had DMEK (*n* = 12, 75%) and those who did not (*n* = 4, 25%). Statistical analysis showed significantly better BCVA in the deferred group at 1-month post-cataract surgery, compared to the group that went on to have DMEK (Table 3, 0.21 ± 0.21 vs. 0.86 ± 0.29 LogMAR, *P* = 0.004). The other parameters, including baseline BCVA and CCT at any time point documented, and BCVA or CCT change (either pre-DMEK or at 6 months, compared to baseline), were not statistically different. With baseline BCVA as the independent variable, and DMEK/non-DMEK groups as dependent variables, the ROC curve analysis (Fig. 2) showed AUROC of 0.841, with the optimal cutoff threshold for predicting DMEK/non-DMEK group as 0.44 LogMAR (sensitivity 81.8% and specificity 75%). Cataract grading is shown in Table 3. The four patients with improved visual acuity had cataract classifications of NO5NC5, NO4NC4, C4 and C4P4, respectively.

**Table 3:** Subgroup analysis: In the case group, those who proceeded to DMEK after cataract surgery and those who did not.

	DMEK group (*n* = 12)^a^	Non-DMEK group (*n* = 4)^a^	*P*^b^
Age (years)	69.75 ± 5.93	62.75 ± 11.32	0.316
Female, % (n)	91.7 (11)	50 (2)	0.064
BCVA at baseline (logMAR)	0.71 ± 0.31	0.41 ± 0.09	0.078
BCVA at 1 month (logMAR)	0.86 ± 0.29	0.21 ± 0.21	0.004*
BCVA pre-DMEK^c^ (logMAR)	0.79 ± 0.39	0.16 ± 0.05	0.001*
BCVA change pre-DMEK^c^ (logMAR)	0.08 ± 0.51	− 0.25 ± 0.10	0.212
CCT at baseline (µm)	646.50 ± 53.10	657.25 ± 66.89	0.770
CCT at 1 month (µm)	633.78 ± 37.99	670.63 ± 120.70	1.000
CCT pre-DMEK^c^ (µm)	673.75 ± 101.80	636.75 ± 94.93	0.684
CCT change pre-DMEK^c^ (µm)	27.25 ± 82.75	-20.50 ± 53.44	0.262
Cataract classification (LOCS III), *n*			NA
C3NO5NC5	1	0	
C3NO4NC4	1	0	
C3NO3NC3P3	1	0	
NO5NC5	5	1	
NO4NC4	3	1	
C4	1	1	
C4P4	0	1	

^a^All values are presented as mean ± SD unless otherwise noted.

^b^Mann-Whitney *U* test.

^c^CCT and BCVA documented at 6 months if DMEK surgery was deferred.

BCVA, best corrected visual acuity; CCT, central corneal thickness; DMEK, Descemet’s membrane endothelial keratoplasty.

## Discussion

This study had two key findings. First, for FECD patients with decompensated corneas awaiting transplantation, phacoemulsification surgery did not lead to significant progression of CCT nor deterioration of visual acuity. Although CCT does not directly equal the degree of corneal decompensation, there are no other surrogate markers except endothelial cell count, which was indecipherable on specular microscopy in all of our patients, due to preexisting cell loss and presence of guttae. In conditions where specular microscopy results are unobtainable, CCT can be used to indirectly measure endothelial function^7,16^. Baseline CCT was 690.53 ± 106.74 µm in the control group and 649.19 ± 54.65 µm in the case group (*P* = 0.338), reflecting similar severity of decompensation between groups. At 1-month after cataract surgery, CCT in the case group (645.12 ± 70.13 µm) had returned to baseline values. In comparison, in corneas with preserved endothelial cell counts (ranging from 1800 to 3000/mm^2^), previous studies demonstrated that CCT returned to pre-cataract surgery values after 1–3 months (mean 539–542.9 µm), with endothelial cell loss of 16% after surgery^5,17^. Thus far, no previous published data on cataract surgery in the decompensated cornea was found on literature review, possibly because managing the cataract first, the surgeon faces the predicament of performing an intraocular procedure through a cloudy cornea with poor visibility^18,19^.

The second key finding was that 25% of patients in the case group regained significantly better vision post-cataract surgery to the point that endothelial keratoplasty was deferred (0.21 ± 0.21 LogMAR at 1-month). All patients had clinically significant cataracts with gradings shown in Table 3. Similar to our findings, Birbal et al.^20^ observed that 30% of eyes with concomitant moderate to advanced cataract and FECD that received cataract surgery first were pleased with the visual outcome and did not require subsequent corneal transplantation, although they did not specify the severity of visual impairment and corneal decompensation. Seitzman et al*.*^21^ reported that 83% of patients with FECD and corneal thickness ≥ 640 µm did not need a corneal transplant within the first year after cataract surgery, and had an average postoperative BCVA of 20/50 (0.4 LogMAR). In contrast to our study, they excluded patients with preoperative slit-lamp evidence of corneal decompensation, in whom triple procedures were performed instead^21^. This discrepancy in inclusion criteria could explain why most of our patients still went on to receive DMEK. Regarding cataract type and visual acuity, increasing severity of nuclear and posterior subcapsular opacity had a more detrimental effect on vision, and mixed cataract caused greater reduction in visual acuity than single type cataract^22–24^. In the present study, there was a high percentage of moderate to severe nuclear or posterior subcapsular opacity in both subgroups, 91.7 and 75% in the DMEK and non-DMEK groups, respectively. Both groups had 25% of mixed cataracts. Although it would seem straightforward that eyes with concomitantly worst cataract would appreciate improved visual acuity, the high percentage of moderate to severe cataracts did not translate to an equally large amount of patients with satisfactory vision. It is noteworthy that non-DMEK patients and those who went on to receive DMEK had similar baseline BCVA, baseline CCT and also post-cataract surgery CCT, indicating that the severity of cataract and corneal decompensation were comparable between the two subgroups. Due to the findings that age, gender, baseline CCT and BCVA, and cataract type/severity did not differ significantly between the two subgroups, we tried to identify characteristics that could predict which patients would benefit from cataract surgery alone. After calculating the AUROC, we found baseline BCVA cutoff value 0.44 LogMAR to be a potential predictor to differentiate between DMEK and non-DMEK patients.

Management of corneal decompensation with concomitant clinically significant cataract requires combined or staged endothelial keratoplasty and cataract extraction procedures. Previous literature has shown evolution in evidence during the past decade. Initially, triple DMEK was not associated with a higher risk of complications compared to DMEK alone, and was advocated due to rapid visual recovery with reduced risks and costs^8^. Afterwards, recommendations shifted towards staged surgery with DMEK followed by cataract surgery, in order to achieve the best final visual and refractive results, since the refractive predictability of combined surgery in FECD eyes were inferior to that of cataract surgery alone in eyes with normal corneas^25^. The advantages of DMEK alone over triple DMEK include lower risk of hyphema from intraoperative iridotomy^26^ and less endothelial cell loss^27^. Shahnazaryan et al*.* found that triple DMEK resulted in significantly greater loss of endothelial cells compared to DMEK alone at both 1 month (35% vs. 25%) and 1 year (41% vs. 33%) follow up^27^. Recently, Moshirfar et al.^7^ devised a decision-making tree regarding preoperative considerations for diagnosis of FECD with cataract. They suggest those with CCT > 640 um, and endothelial cell density lower than 1000cells/mm^2^ avoid cataract surgery alone, but those with densities higher than 1000 cells/mm^2^ can consider cataract surgery, albeit recommending triple or staged procedures^7^. Nonetheless, some surgeons prefer to address the cataract first and subsequently perform an endothelial keratoplasty^20,28^. As aforementioned, Birbal et al.^20^ observed that some patients were satisfied with their vision after simply cataract removal and did not require DMEK. This allowed postponing corneal transplantation while eliminating post-operative care, such as regular follow ups after keratoplasty and lifelong topical steroid use^20^. Moreover, cataract surgery alone can be arranged at any time, while corneal transplantation is dependent on availability of donor tissue^11^. Triple procedure is only possible in countries with abundant supply of donor corneas, and in many developing countries, the supply cannot meet the demand^4,9^.

There are some limitations of the current study that should be addressed. First, the present study was retrospective in design with a relatively small sample size. Nevertheless, post-hoc power analyses for the subgroups showed ≥ 85% power based on the primary endpoints of BCVA. Further prospective research with a larger sample size is needed to confirm our findings, as well as elucidate other predictors for patients who have the chance of improving visual acuity after cataract surgery. Second, it is difficult to compare head-to-head the pre-operative severity of cataracts and to what extent they contribute to decreased visual acuity. However, this drawback is inherent in cataract research. Another limitation is that we did not evaluate the intraoperative phaco energy and time, which would influence the endothelium during cataract surgery. Despite these limitations, the present results are relevant when considering treatment of concomitant corneal decompensation and moderate to severe cataracts in clinical practice, notably due to the number of patients waitlisted for a donor cornea.

In conclusion, when corneal donors are not widely available, cataract surgery can be considered in patients with corneal decompensation awaiting endothelial keratoplasty. Statistically, the current study demonstrated that patients did not experience further deterioration in corneal thickness nor visual acuity after phacoemulsification. Some patients had the chance to attain satisfactory visual acuity and postpone corneal transplantation.
